# Research on temperature prediction method for rail transit train inverters based on spatial and timing improving Transformer

**DOI:** 10.1038/s41598-025-19004-8

**Published:** 2025-09-29

**Authors:** Yanhong Feng, Yinan Zhai, Fu Wang, Xunran Yu, Jinglei Li, Xinyu Chen

**Affiliations:** 1 School of Information Science & Engineering, Dalian Ocean University, Dalian, China; 2 Modern Educational Technology Center, Dalian Vocational Technical College, Dalian, China; 3 School of Civil Engineering and Architecture, Wuhan Institute of Technology, Wuhan, China; 4 Transport Planning and Research Institute Ministry of Transport, Beijing, China; 5 International Navigation College, Hainan Tropical Ocean University, Hainan, China

**Keywords:** Equipment temperature prediction, Generative adversarial networks, Random forest, Transformer, Model fusion, Civil engineering, Electrical and electronic engineering

## Abstract

Inverter overheating is a critical fault factor in rail transit systems. To address the challenges of sparse low-voltage data and high-dimensional input features, we propose a hybrid prediction framework for inverter temperature. The Random Masked Dual DCGAN (RTDG) model is introduced to enhance low-voltage data diversity, while a Gaussian Markov Random Field (GMRF) method performs dimensionality reduction by identifying key variables. To capture spatio-temporal dependencies, an enhanced Transformer architecture (STTr) is constructed, integrating state space modeling and temporal normalization. These components are fused using a weighted stacking strategy. The model is trained and validated on real-world rail transit datasets. Performance is evaluated using MSE, RMSE, and MAE metrics. Experimental results show that the proposed model outperforms conventional approaches, achieving a 4.93% improvement over single models and a 9.73% gain compared to non-augmented training. This framework supports intelligent fault prevention and contributes to the safe, efficient operation of modern rail systems.

**Supplementary Information:**

The online version contains supplementary material available at 10.1038/s41598-025-19004-8.

## Introduction

With the rapid development of modern cities, urban rail transit has become an essential public transportation component due to its safety, efficiency, and sustainability^1^. As systems grow in scale and complexity, the reliability of key components like inverters—responsible for DC–AC conversion in traction systems—has garnered increasing attention^2^. Temperature abnormalities in traction inverters are a primary cause of degradation and fault^3^. Real-time monitoring and accurate forecasting of inverter temperature can enable early fault warnings, reduce maintenance costs, and improve operational safety^4^. However, traditional statistical or physical modeling methods struggle to capture nonlinear relationships in high-dimensional sensor data and lack generalization to new contexts^5^. Deep learning has emerged as a powerful tool for modeling complex temporal patterns in multivariate data^6^. Among architectures, Transformers excel in sequence modeling via self-attention, which captures long-range dependencies^7^. Variants like FEDformer^8^ enhancing temporal modeling through frequency decomposition inform our spatio-temporal fusion design, with such models showing state-of-the-art performance in energy and climate prediction, supporting their potential for industrial temperature forecasting^9^. To address sparse fault and low-voltage data, generative models like GANs have been used for synthetic data generation^10^. Advanced architectures (e.g., Wasserstein GAN, Coupled GAN) extend GANs to domain-specific signals^11^, and GAN-based data augmentation mitigates class imbalance, improving model generalization under rare conditions^12^. However, augmentation increases feature dimensionality, risking noise and overfitting. Thus, effective feature selection is critical: ensemble methods like Random Forest provide interpretable importance scores^13^, while techniques such as ST-Norm^14^ and GMRF-based filtering isolate informative features from noise^15^. In railway predictive maintenance, spatial–temporal learning has advanced, with models like STTRE^16^ and PDFormer^17^ integrating spatial layout and time-series dynamics to improve prediction. Attention-based anomaly detection frameworks also succeed in real-time monitoring of temperature spikes and fault precursors^18^.

In summary, while Transformers offer strong temporal modeling, their performance can be enhanced by improving input quality via GAN-based augmentation and feature compression. This study proposes a spatial–temporal enhanced Transformer framework integrating Random Masked Dual DCGAN (RTDG) for data enrichment and parallel GMRF-based feature selection, evaluated on real rail transit inverter datasets to support early fault detection and operational safety.To the best of our knowledge, this is the first attempt to integrate generative data augmentation, parallel feature filtering, and spatio-temporal attention modeling into a unified framework for inverter temperature prediction in rail transit systems.

## RTDG-GMRF enhanced feature extraction

This study proposes a two-stage feature processing framework to address data imbalance and high dimensionality in inverter temperature prediction. The approach consists of an improved data augmentation model (RTDG) and a dimensionality reduction module based on Random Forest with parallel GMRF optimization.

### RTDG-based low-voltage data enhancement

To enrich sparse low-voltage signals in minority classes, we develop the Random Masked Dual DCGAN (RTDG) model. It extends the conventional DCGAN by integrating a Random Masked Data (RMD) block and a Two-way Adversarial Discriminator (TAD).

The RMD module partitions input data into subregions and randomly masks each region to inject variability, enhancing robustness against incomplete signals. TAD comprises two parallel discriminators: one observes original sequences, while the other processes transposed data, reducing distribution bias in generated samples by dual-perspective discrimination, enabling diverse discriminative perspectives. During training, minority-class samples are selectively upsampled, noise vectors are injected, and adversarial training is conducted with iterative backpropagation until convergence. The final generator produces augmented samples for subsequent prediction modeling.

**Fig. 1:**
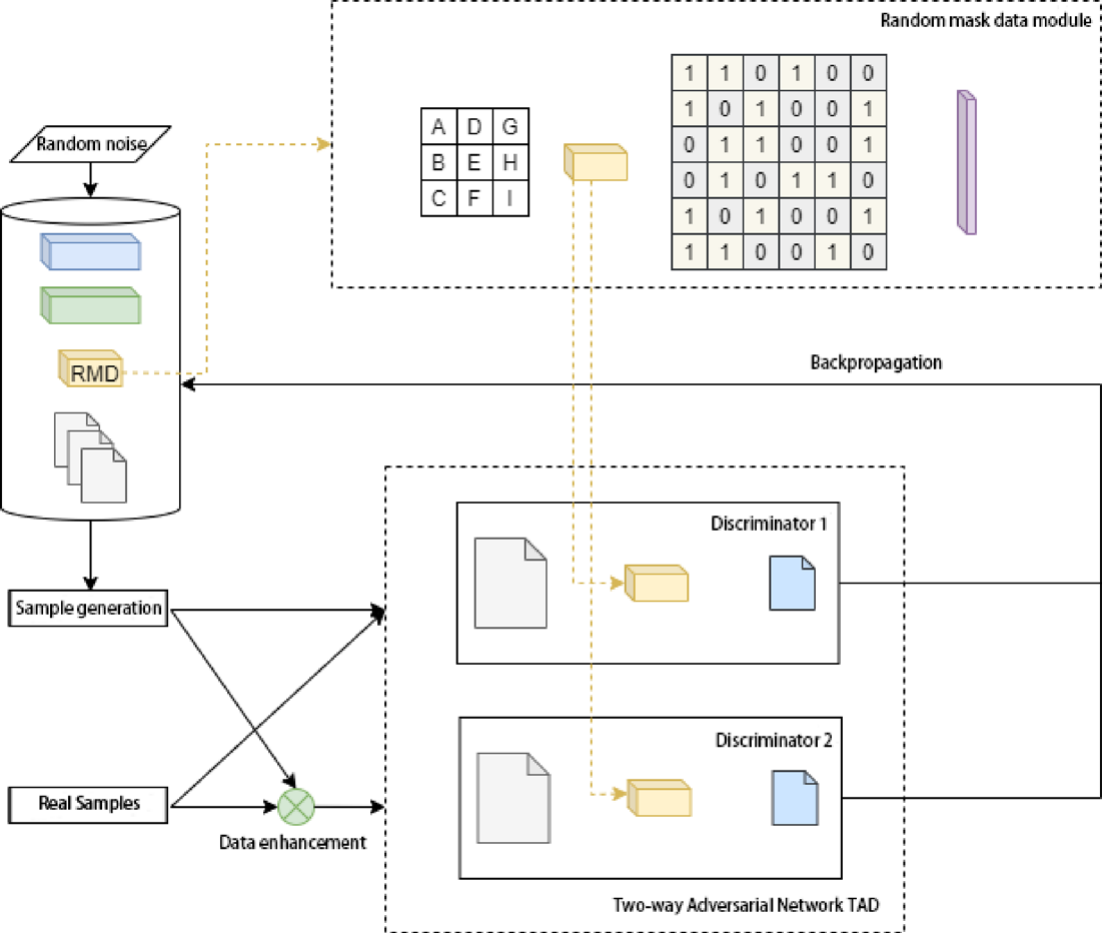
Architecture of the RTDG data enhancement module.

This enhancement significantly increases data diversity, helping the model generalize across complex operational conditions. The architecture of the RTDG module supporting this enhancement is illustrated in Fig. 1.

### GMRF-based dimensionality reduction

Although data augmentation improves representation diversity, it also increases feature space dimensionality. To alleviate computational complexity and reduce model overfitting, a Random Forest-based feature selection method is employed, further optimized using a parallel GMRF structure.

Each feature’s relevance is evaluated based on its average contribution to impurity reduction across the forest. The importance score of feature ${\text{j}}$ is calculated by averaging its contribution to impurity reduction across all decision trees, as shown in Eq. (1):

1 $${\text{Importance}}\left( {\text{j}} \right) = \frac{1}{{\text{N}}}\mathop \sum \limits_{{{\text{i}} = 1}}^{{\text{N}}} \mathop \sum \limits_{{{\text{t}} \in {\text{T}}_{{{\text{i}}\left( {\text{j}} \right)}} }}^{{}} \Delta {\text{I}}_{{{\text{it}}}}$$

where ${\text{T}}_{{\text{i}}} \left( {\text{j}} \right)$ is the set of nodes in tree i that use feature j, and $\Delta {\text{I}}_{{{\text{it}}}}$ is the impurity reduction at node ${\text{t}}$. Parallel processing using a MapReduce-like framework allows fast computation over large-scale rail transit datasets. This integrated RTDG-GMRF pipeline ensures both data richness and feature compactness, providing high-quality input for the downstream temperature prediction model.

To evaluate the RTDG-GMRF framework, we utilized historical inverter data collected from urban rail vehicles operating in a Chinese city over the past 5 years. The dataset contains over 2 million entries across 59 CSV files, encompassing electrical signal records, status indicators, and fault codes. After preprocessing (including outlier removal, Min–Max normalization, and One-Hot encoding for categorical fields), the enhanced dataset was divided into training and test sets in an 8:2 ratio.

Following augmentation and feature selection, the top-ranked indicators for inverter temperature prediction included: SIVCLP life signal (importance: 0.193), output U-phase current (0.107), and battery temperature (DDU) (0.100). SIVCLP life signal reflects inverter aging, while output U-phase current correlates with real-time load, both being core factors affecting temperature, validating feature selection rationality. Key features identified through ranking (as shown in Table 1) validate the GMRF-based dimensionality reduction module’s ability to preserve informative variables for downstream modeling. These features were selected based on their close correlation with inverter temperature variations and their relevance to thermal degradation, load response, and environmental heating patterns. This confirms that the model input selection process is directly dependent on temperature-related characteristics.

**Table 1:**
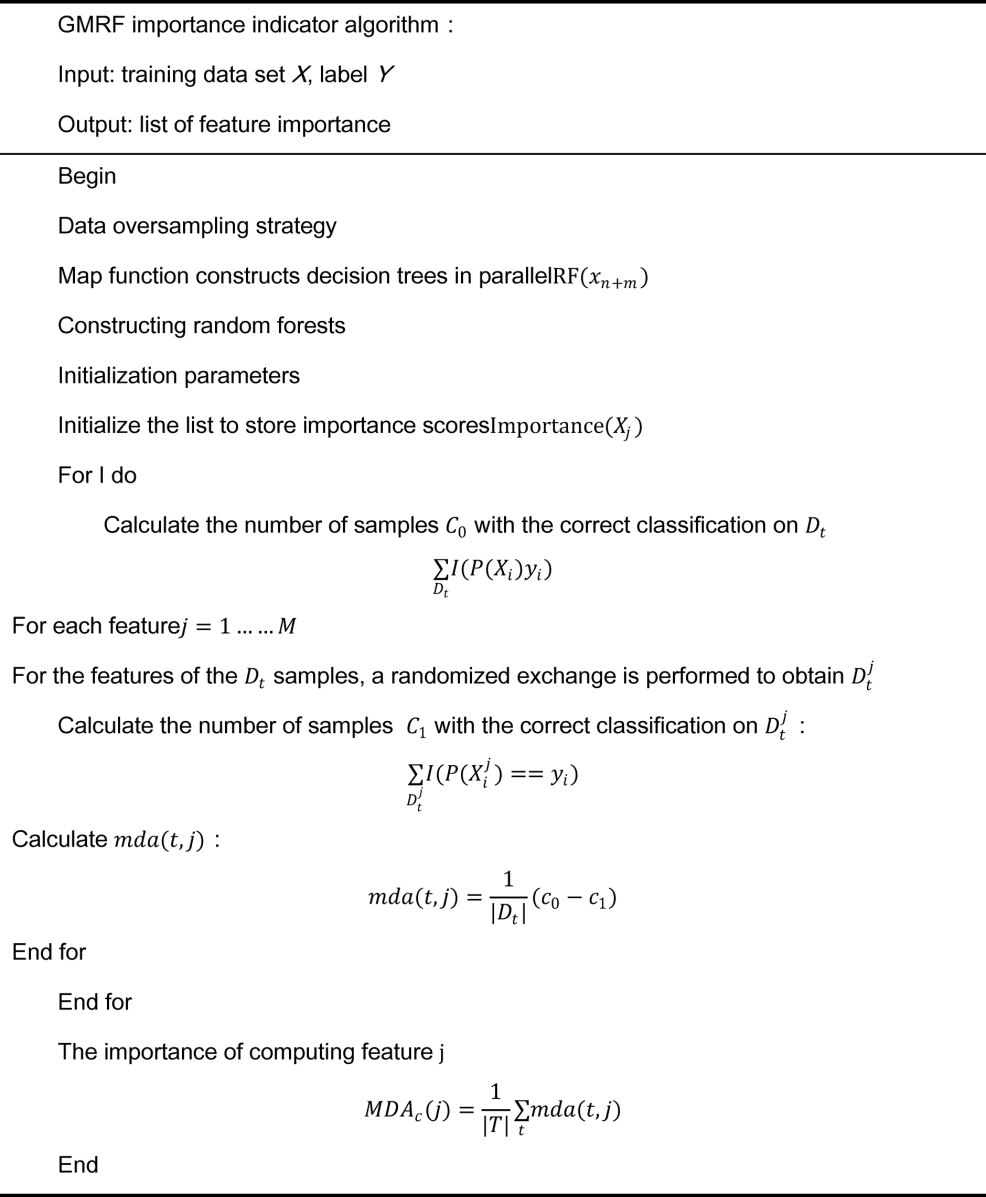
GMRF importance index algorithm.

## Transformer-based temperature prediction model (STTr)

To improve temperature prediction accuracy under complex operating conditions, this study proposes a Transformer-based model named STTr (State-Space and Temporal-enhanced Transformer). STTr enhances traditional Transformer by integrating state-space modeling and temporal attention refinement. The outputs of both branches are fused via weighted stacking, forming a unified spatial–temporal prediction framework.

### Overall architecture and fusion strategy

The STTr architecture consists of two attention-based branches that independently extract spatial and temporal features from input sequences. Their prediction results are integrated through a weighted fusion mechanism. The final output ${\hat{\text{y}}}$ is calculated based on the predictions of both branches, as shown in Eq. (2):

2 $$\hat{y} = \alpha_{1} y_{1} + \alpha_{2} y_{2}$$

where ${\text{y}}_{1}$ and ${\text{y}}_{2}$ represent the outputs of the spatial and temporal branches respectively, and the fusion weights $\alpha_{1}$ and $\alpha_{2}$ satisfy $\alpha_{1} + \alpha_{2} = 1$. The values of the weights are adaptively assigned according to validation performance on the dataset.

**Fig. 2:**
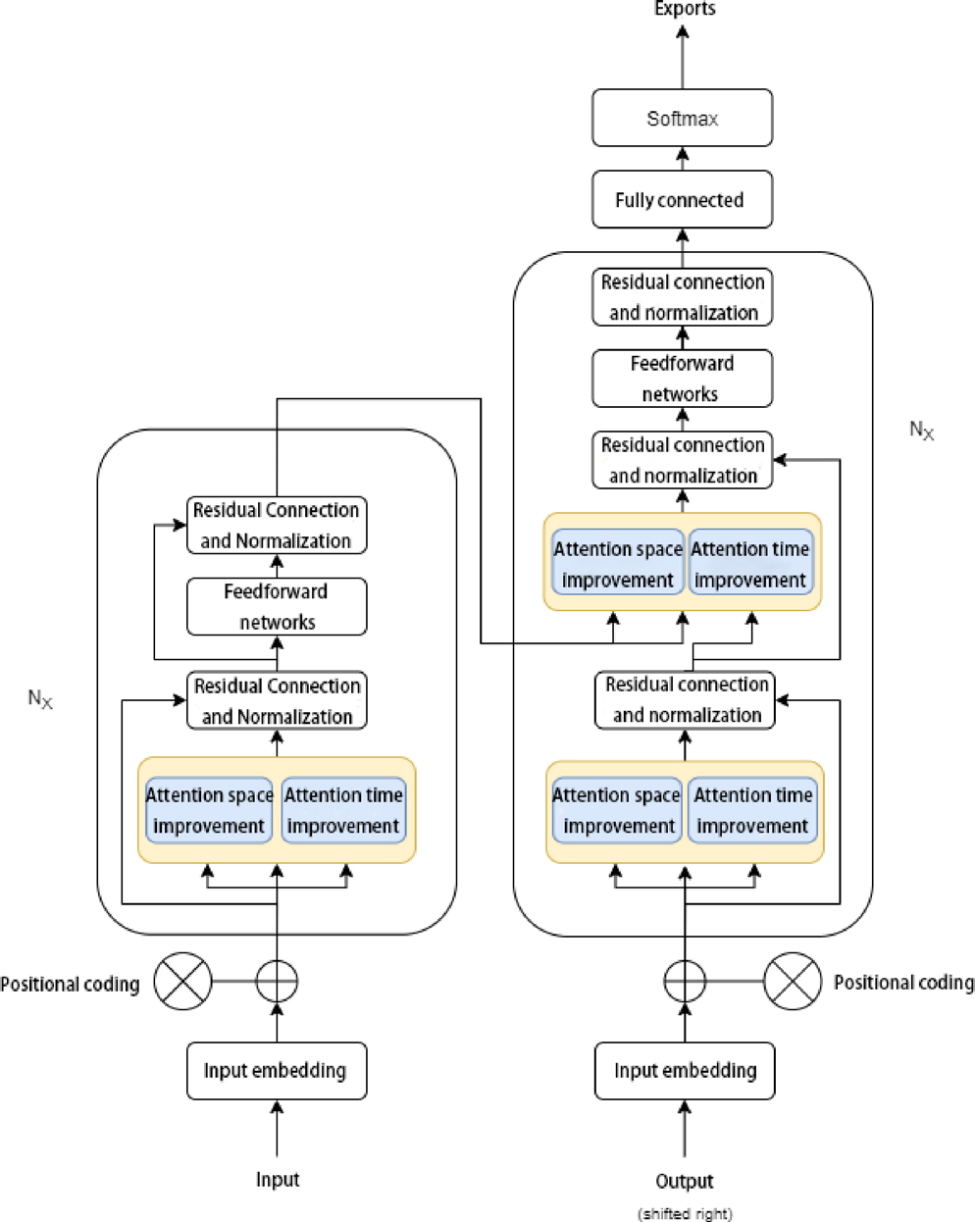
STTr model architecture with spatial–temporal attention fusion.

This dual-branch ensemble structure mitigates the limitations of single-model dependency and enhances generalization across varying signal conditions. The spatial–temporal attention fusion design of the STTr model is visualized in Fig. 2.

### Spatial feature modeling via state space module (SSM)

The state-space modeling branch introduces hidden state variables to capture latent structural patterns in the reduced-dimensional input data. The system state ${\text{h}}_{{\text{t}}}$ at time ${\text{t}}$ evolves recursively based on the current state and control input. The update process follows the state transfer rule, as shown in Eq. (3):

3 $$h_{t + 1} = Ah_{t} + Bu_{t} + \omega_{t}$$

The output prediction ${\text{y}}_{{\text{t}}}$ is derived from the hidden state, as shown in Eq. (4):

4 $$y_{t} = Ch_{t} + v_{t}$$

In the above equations, ${\text{A}}$, ${\text{B}}$, and ${\text{C}}$ are learnable transformation matrices, ${\text{u}}_{{\text{t}}}$ represents the control input (optional), while ${\upomega }_{{\text{t }}}$ and ${\text{v}}_{{\text{t}}}$ are zero-mean Gaussian process noise and observation noise respectively. This formulation enables the model to capture long-range interactions and smooth fluctuations by modeling internal dynamics. To further enhance spatial stability, a spatial normalization layer is applied to balance local and global feature distributions.

### Temporal feature modeling via normalized attention (TN)

In the temporal branch, attention mechanisms are used to refine the significance of time steps within the sequence. Given a hidden state sequence $\left\{ {{\text{h}}_{1} ,{\text{h}}_{1} ,..,{\text{h}}_{{\text{T}}} } \right\}$, the attention score for each time step is computed using a non-linear transformation, as shown in Eq. (5):

5 $$e_{t} = \tan h\left( {Wh_{t} + b} \right)$$

The normalized attention weights ${\upalpha }_{{\text{t}}}$ are obtained using the softmax function, as shown in Eq. (6):

6 $$\alpha_{t} = \frac{{exp\left( {e_{t} } \right)}}{{\mathop \sum \nolimits_{K = 1}^{T} exp\left( {e_{k} } \right)}}$$

Here, ${\text{W}}$ and b are learnable parameters. The final output of the temporal attention module is calculated as the weighted sum of all hidden states. This design allows the model to dynamically emphasize the most relevant time points and suppress redundant noise in long sequences.

### Model training and optimization strategy

The STTr model is trained in an end-to-end manner using maximum likelihood estimation. During training, the objective is to minimize the discrepancy between actual and predicted inverter temperatures using the Mean Squared Error (MSE) loss. Root Mean Squared Error (RMSE) and Mean Absolute Error (MAE) are also employed to evaluate model performance on unseen data.

To ensure convergence, the learning rate is controlled using the Adagrad algorithm with adaptive decay. Grid search is applied to tune key hyperparameters, including learning rate, number of hidden units, batch size, time steps, and dropout ratio. Early stopping is introduced to avoid overfitting, terminating training when no improvement is observed on the validation set. Under these configurations, the model typically achieves convergence within 120 training epochs.

## Experimental design and model evaluation

### Experimental setup and model variants

To assess the performance of the proposed model, five comparative models (M1–M5) were designed, each representing a different combination of temporal and spatial modules. As shown in Table 2, the models are categorized by their architectural components and functional enhancements.

**Table 2:** Model configurations and architectural differences.

Model	Structure	Improvements
M1	Tr-DC	Baseline Transformer with gating mechanism
M2	Tr-DC-TN	M1 + temporal normalization module
M3	Tr-TC	Baseline Transformer with trend coding
M4	Tr-TC-SSM	M3 + state-space modeling
M5	Tr-WF (STTr)	M2 + M4 fusion via weighted stacking

All models were trained under identical hyperparameter settings and received the same RTDG-GMRF enhanced inputs to ensure fair comparison.

### Evaluation metrics

Three standard error metrics were used to evaluate prediction accuracy: Mean Squared Error (MSE), Root Mean Squared Error (RMSE), and Mean Absolute Error (MAE). Their definitions are given as shown in Eqs. (7)–(9):

7 $${\text{MSE}} = \frac{1}{{\text{n}}}\mathop \sum \limits_{{{\text{i}} = 1}}^{{\text{n}}} ({\text{y}}_{{\text{i}}} - {\hat{\text{y}}}_{{\text{i}}} )^{2}$$

8 $${\text{RMSE}} = \sqrt {\frac{1}{{\text{n}}}\mathop \sum \limits_{{{\text{i}} = 1}}^{{\text{n}}} \left( {{\text{y}}_{{\text{i}}} - {\hat{\text{y}}}_{{\text{i}}} } \right)^{2} }$$

9 $${\text{MAE}} = \frac{1}{{\text{n}}}\mathop \sum \limits_{{{\text{i}} = 1}}^{{\text{n}}} \left| {{\text{y}}_{{\text{i}}} - {\hat{\text{y}}}_{{\text{i}}} } \right|$$

where ${\text{y}}_{{\text{i}}}$ and ${\hat{\text{y}}}_{{\text{i}}}$ denote the ground truth and predicted inverter temperatures for the ${\text{i}}$-th sample.

### Data partitioning and processing flow

To ensure fair model evaluation and reduce performance bias, the original dataset was partitioned into three subsets: 80% for training, 10% for validation, and 10% for testing. During preprocessing, outliers were removed using interquartile filtering, numerical features were normalized using Min–Max scaling, and categorical variables were transformed via one-hot encoding.

The experimental pipeline adheres to a unified modeling protocol in which all models receive identically preprocessed inputs and are trained, validated, and tested under consistent conditions. This uniform treatment guarantees that differences in performance metrics are attributable solely to architectural distinctions between models, thus ensuring reproducibility and comparability of the experimental results.

### Model comparison and performance validation

**Fig. 3:**
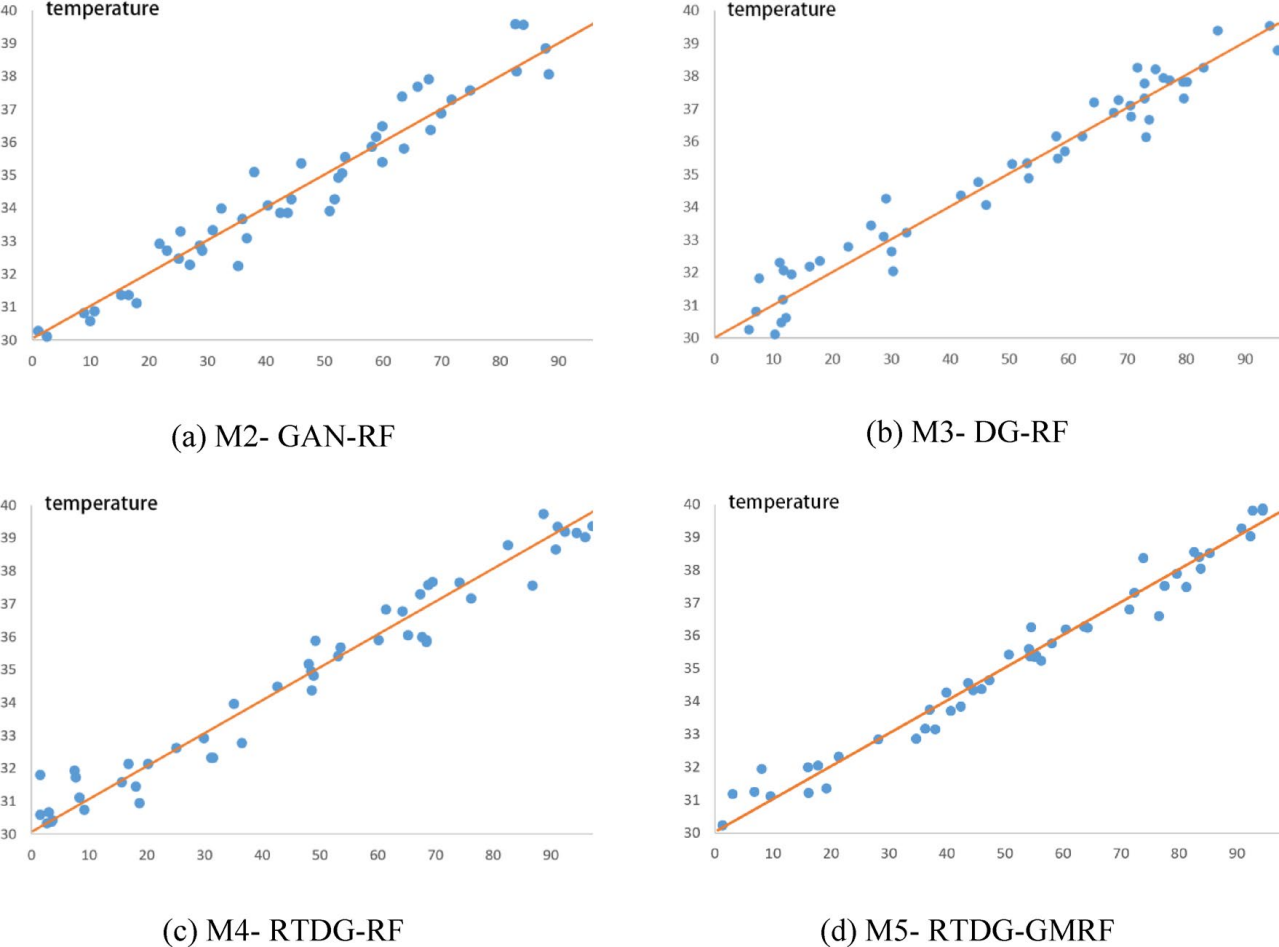
Inverter temperature predictions of M1–M5 compared to ground truth. Note: (**a**)–(**d**) correspond to comparison models in the data augmentation and dimensionality reduction stage (M2-GAN-RF, M3-DG-RF, M4-RTDG-RF, M5-RTDG-GMRF), validating the RTDG-GMRF framework. M1-M5 in Table 1 refer to Transformer variants in the downstream temperature prediction stage, representing preprocessing and prediction phases respectively.

Prediction results of the five models are compared against the actual temperature curve. As shown in Fig. 3, the M5-STTr model aligns closely with true values, especially during sharp temperature transitions.

**Fig. 4:**
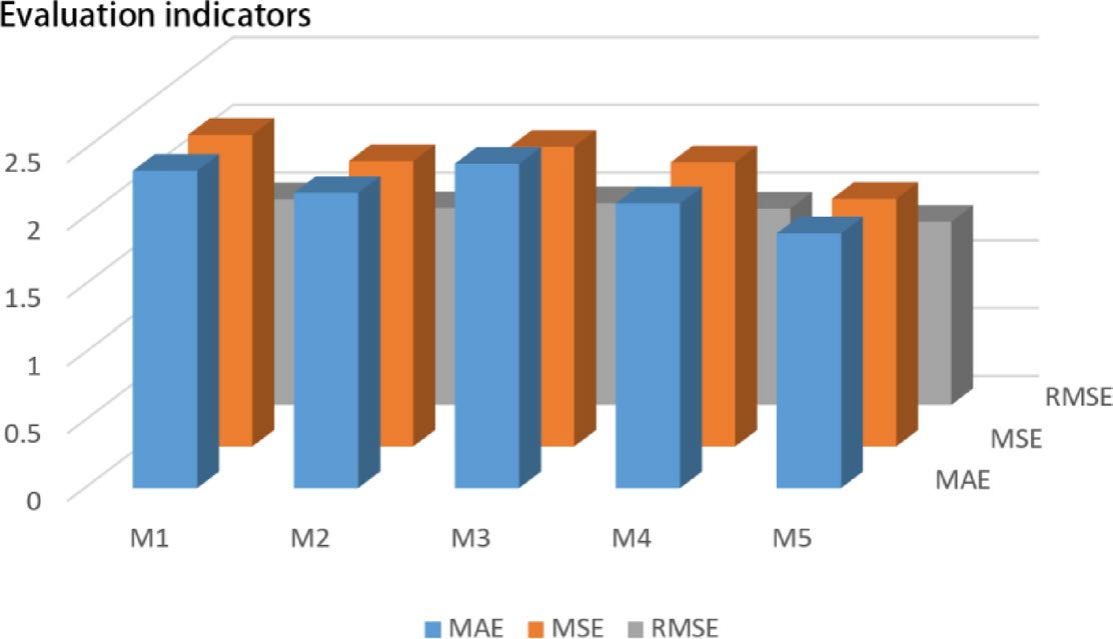
Error metrics of M1–M5 models on test set. Note: MSE unit is °C^2^; RMSE and MAE units are °C. Lower values indicate higher prediction accuracy.

Quantitative comparisons of test set performance are illustrated in Fig. 4, showing M5 achieved the lowest error across all metrics. On the test set, M5 reduced MSE by 4.93% compared to M1 (single-model baseline); compared to a non-augmented counterpart (same architecture without RTDG), M5 reduced MSE by 9.73%, aligning with the core findings.

These results confirm that combining spatial and temporal mechanisms via weighted fusion significantly enhances model generalization and robustness compared to single-path designs.

## Robustness analysis and disturbance evaluation

To validate the practical reliability of STTr in real-world applications, a set of robustness tests was designed under two typical disturbance scenarios: (1) noisy signal injection and (2) feature perturbation testing. The goal was to assess the model’s resistance to data degradation and evaluate its performance stability across dynamic environments.

### Gaussian noise resistance test

**Fig. 5:**
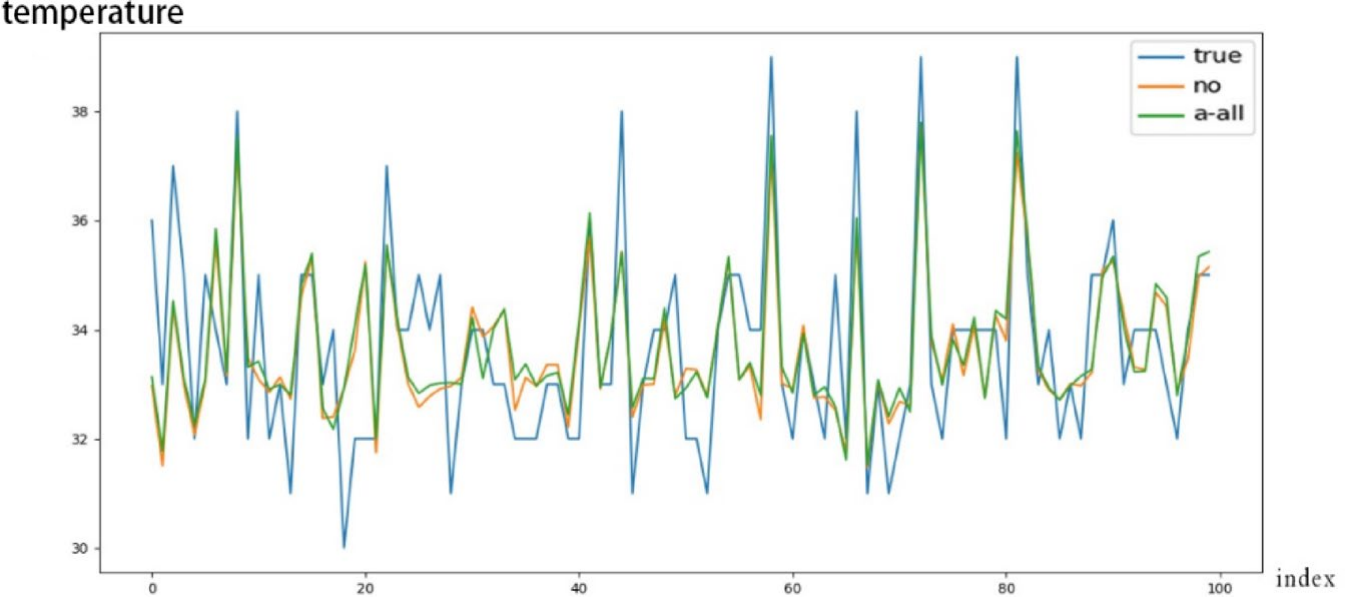
Error metrics comparison under Gaussian noise and drift interference.

The first test introduces artificial noise to simulate sensor uncertainty. Gaussian noise with standard deviation ${\upsigma } = 0.2$ was injected into randomly selected channels, and long-term drift was added to mimic sensor aging. As shown in Fig. 5, the baseline model M1 exhibited significant error growth, while STTr (M5) maintained the lowest error, verifying its stability against noise.

STTr’s dual-path architecture enables compensation between spatial and temporal cues, allowing it to resist irregular signal corruption more effectively than single-branch structures.

### Feature perturbation test

**Fig. 6:**
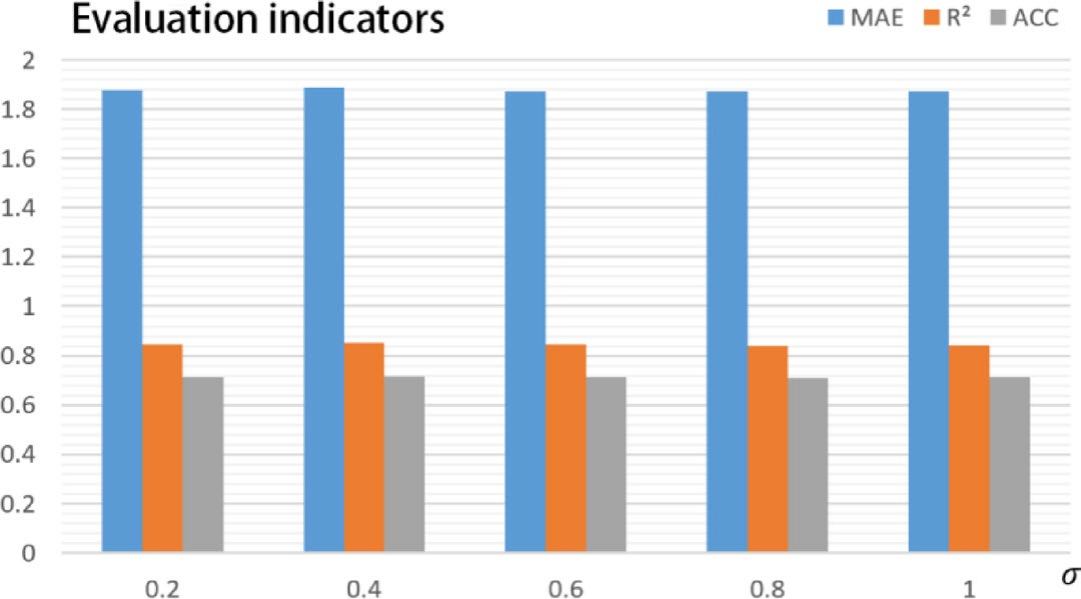
Error variation under progressive feature corruption. Note: ${\sigma }$ denotes feature perturbation intensity (0 → 1 corresponds to random masking ratio of high-importance features increasing from 0 to 100%). Metrics include MAE (Mean Absolute Error), ${\text{R}}^{2}$ (Coefficient of Determination), and ACC (Accuracy), with values reflecting model tolerance to feature corruption.

The second experiment evaluates model response to missing or corrupted features. High-importance variables (e.g., SIVCLP life signal, output U-phase current) were selectively masked or replaced with noise. As shown in Fig. 6 (error variation under progressive feature corruption), STTr (M5) exhibited the smallest MAE growth and highest ${\text{R}}^{2}$ retention compared to M1–M4, indicating stronger tolerance to key feature corruption.

This result highlights the effectiveness of RTDG-GMRF feature optimization, which prioritizes resilient indicators and improves feature redundancy.

### Robustness mechanism summary

The robustness analysis confirms that the proposed STTr model maintains stable predictive performance under both Gaussian noise and feature perturbation conditions. This resilience is primarily attributed to its dual-branch architecture, which enables mutual compensation between spatial and temporal representations. Additionally, the attention mechanism dynamically reweights inputs to suppress the influence of noisy timestamps, while the GMRF-based feature selection module ensures that the input space is both compact and robust against degradation. These characteristics collectively make STTr more reliable for practical applications involving unstable or noisy rail transit monitoring data. These robustness experiments demonstrate that the synthetic samples generated by RTDG do not introduce harmful artifacts, and the model remains stable under noise and feature corruption. This supports the validity of RTDG-based augmentation in practical settings.

## Conclusion

This study proposed a novel temperature prediction framework for rail transit inverter systems based on a spatial–temporal Transformer (STTr) architecture. To address data imbalance and redundancy, a two-stage preprocessing pipeline combining Random Masked Dual DCGAN (RTDG) and GMRF-based feature optimization was developed. The resulting input set preserved informative indicators while suppressing noise and dimensionality inflation. Experimental results on real-world data confirmed the superior performance and robustness of the proposed approach. These tests were designed to simulate common fault scenarios in real-world systems, such as sensor drift, environmental noise, and feature degradation due to hardware faults or communication loss.

The main contributions of this study are summarized as follows. A RTDG-GMRF preprocessing framework was established, which effectively enhances data diversity and compresses feature space. A spatial–temporal Transformer (STTr) model was proposed, integrating attention-guided dual-branch learning and adaptive fusion strategies. Extensive experiments under both clean and disturbed scenarios confirmed the accuracy, robustness, and generalization advantages of STTr over existing models.

Unlike previous approaches that treat data augmentation, feature selection, and modeling separately, our framework unifies all three in a coordinated architecture tailored for complex, noisy rail environments. These contributions indicate that the proposed method is well-suited for inverter condition monitoring and predictive maintenance in urban rail transit systems. Future work will focus on optimizing STTr’s inference efficiency for real-time deployment on edge devices in rail transit systems. In particular, techniques such as model pruning, quantization, and lightweight Transformer design will be explored to reduce computational complexity and enhance deployability in edge environments. The generalizability of the model may be influenced by variations in train configurations, route topologies, and external environmental factors. Future studies will focus on validating the framework across multiple cities and rail systems to assess cross-domain adaptability.

## Supplementary Information

Below is the link to the electronic supplementary material.

Supplementary Material 1

## Data Availability

All data generated or analysed during this study are included in this published article.
