# Supplementary figures and images for: Research on temperature prediction method for rail transit train inverters based on spatial and timing improving Transformer
Source: Sci Rep. 2025 Sep 29;15:33607. doi: 10.1038/s41598-025-19004-8 (PMC12480008; doi:10.1038/s41598-025-19004-8)

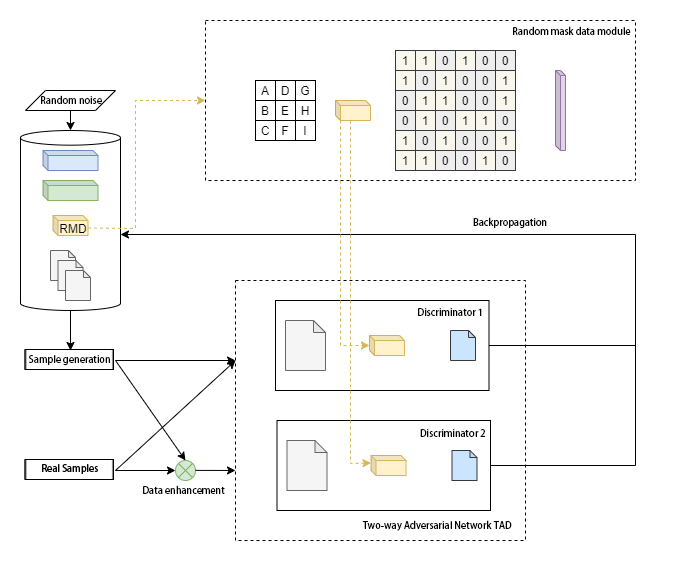

Supplement: Supplementary file 1 — Supplementary Material 1 [file 41598_2025_19004_MOESM1_ESM.zip › figure1.png]

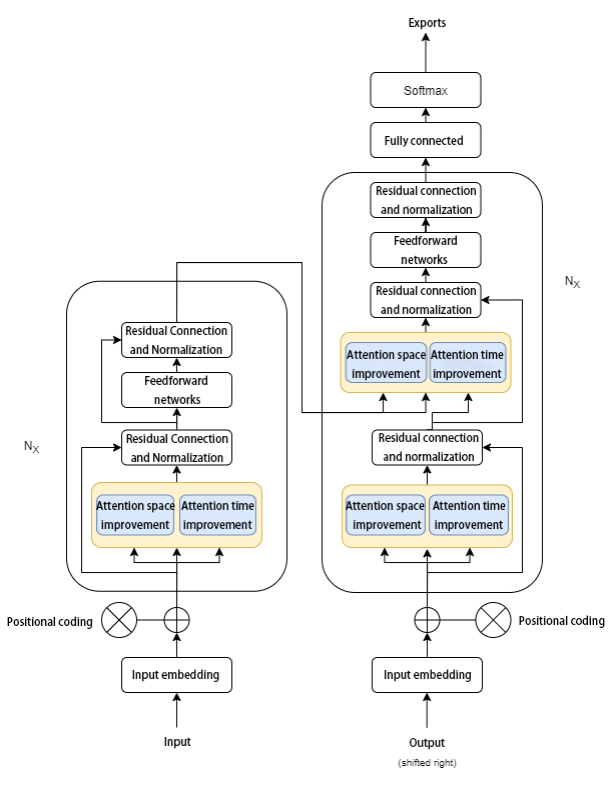

Supplement: Supplementary file 1 — Supplementary Material 1 [file 41598_2025_19004_MOESM1_ESM.zip › figure2.png]

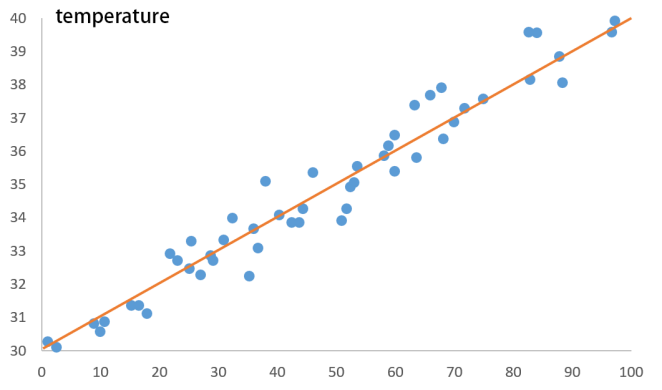

Supplement: Supplementary file 1 — Supplementary Material 1 [file 41598_2025_19004_MOESM1_ESM.zip › figure3(a).png]

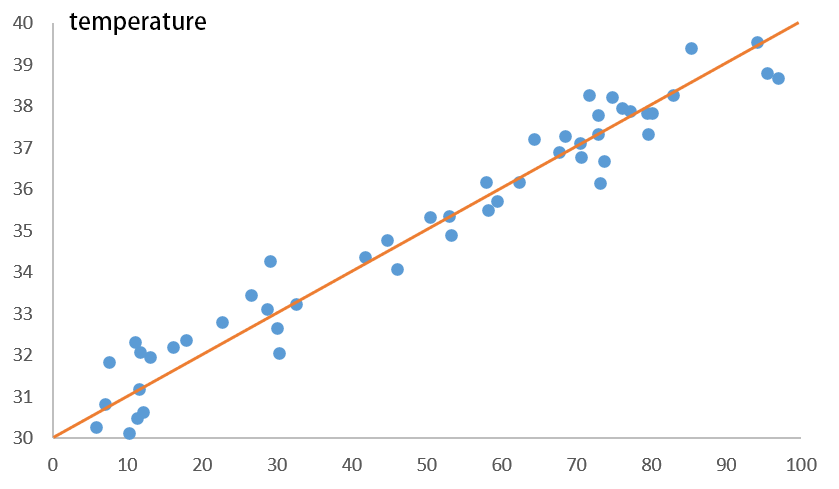

Supplement: Supplementary file 1 — Supplementary Material 1 [file 41598_2025_19004_MOESM1_ESM.zip › figure3(b).png]

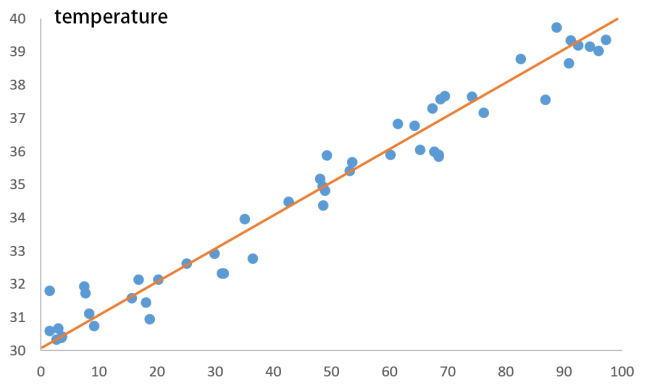

Supplement: Supplementary file 1 — Supplementary Material 1 [file 41598_2025_19004_MOESM1_ESM.zip › figure3(c).png]

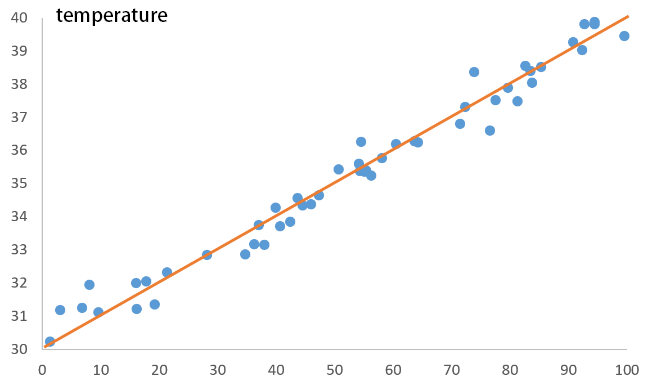

Supplement: Supplementary file 1 — Supplementary Material 1 [file 41598_2025_19004_MOESM1_ESM.zip › figure3(d).png]

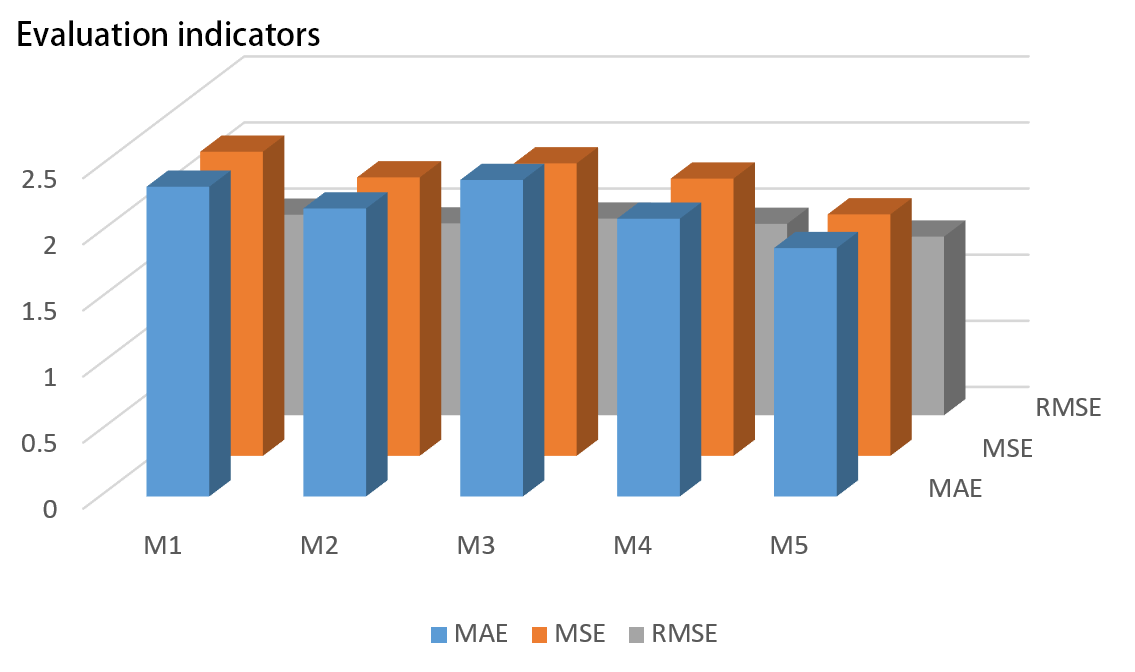

Supplement: Supplementary file 1 — Supplementary Material 1 [file 41598_2025_19004_MOESM1_ESM.zip › figure4.png]

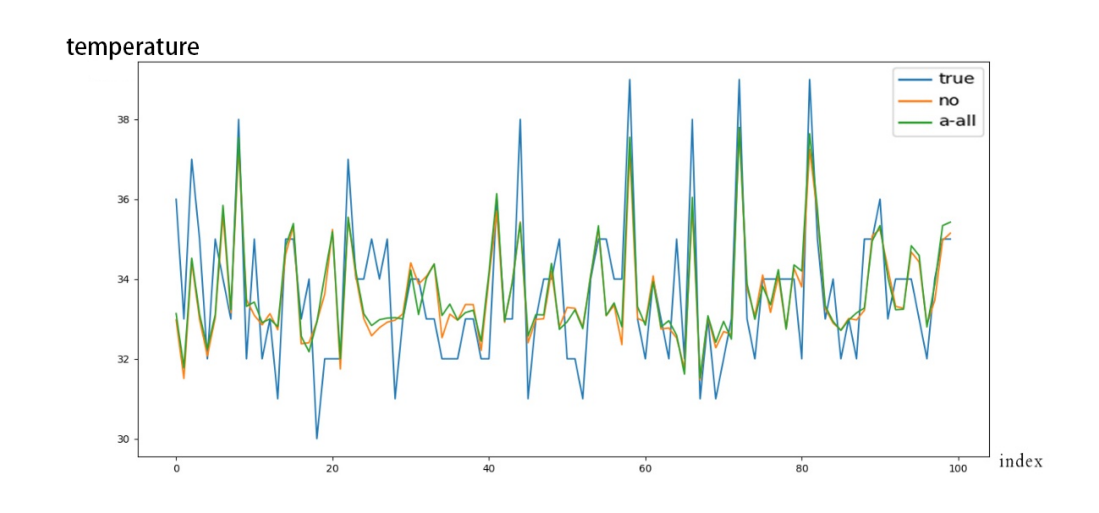

Supplement: Supplementary file 1 — Supplementary Material 1 [file 41598_2025_19004_MOESM1_ESM.zip › figure5.png]

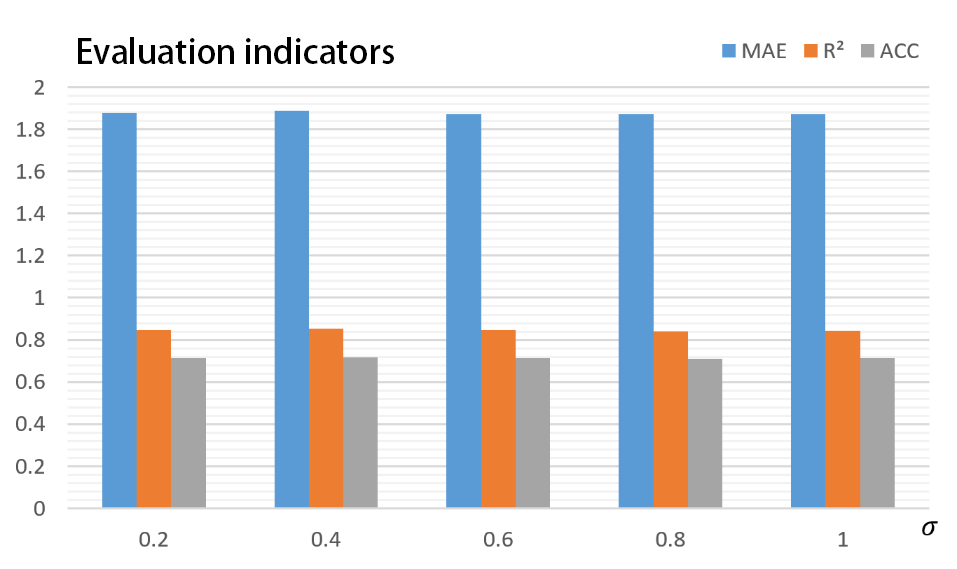

Supplement: Supplementary file 1 — Supplementary Material 1 [file 41598_2025_19004_MOESM1_ESM.zip › figure6.png]
